# How Hydroxyurea Alters the Gut Microbiome: A Longitudinal Study Involving Angolan Children with Sickle Cell Anemia

**DOI:** 10.3390/ijms23169061

**Published:** 2022-08-13

**Authors:** Mariana Delgadinho, Catarina Ginete, Brígida Santos, Carolina Fernandes, Carina Silva, Armandina Miranda, Jocelyne Neto de Vasconcelos, Miguel Brito

**Affiliations:** 1 H&TRC-Health & Technology Research Center, ESTeSL-Escola Superior de Tecnologia da Saúde, Instituto Politécnico de Lisboa, 1990-096 Lisbon, Portugal; 2 Centro de Investigação em Saúde de Angola (CISA), Hospital Geral do Bengo, Bengo 9999, Angola; 3 Hospital Pediátrico David Bernardino (HPDB), Luanda 3067, Angola; 4 Centro de Estatística e Aplicações, Universidade de Lisboa, 1749-016 Lisbon, Portugal; 5 Instituto Nacional de Saúde Doutor Ricardo Jorge (INSA), 1649-016 Lisbon, Portugal

**Keywords:** hydroxyurea, gut microbiome, sickle cell anemia, Angolan children, 16S rRNA

## Abstract

Sickle cell anemia (SCA) is an inherited hematological disorder and a serious global health problem, especially in Sub-Saharan Africa. Although hydroxyurea (HU) is the leading treatment for patients with SCA, its effects on the gut microbiome have not yet been explored. In this context, the aim of this study was to investigate this association by characterizing the gut microbiome of an Angolan SCA pediatric population before and after 6 months of HU treatment. A total of 66 stool samples were obtained and sequenced for the 16S rRNA gene (V3-V4 regions). Significant associations were observed in alpha and beta-diversity, with higher values of species richness for the children naïve for HU. We also noticed that children after HU had higher proportions of several beneficial bacteria, mostly short-chain fatty acids (SCFAs) producing species, such as *Blautia luti*, *Roseburia inulinivorans*, *Eubacterium halli*, *Faecalibacterium*, *Ruminococcus*, *Lactobacillus rogosae*, among others. In addition, before HU there was a higher abundance of *Clostridium_g24*, which includes *C. bolteae* and *C. clostridioforme*, both considered pathogenic. This study provides the first evidence of the HU effect on the gut microbiome and unravels several microorganisms that could be considered candidate biomarkers for disease severity and HU efficacy.

## 1. Introduction

Sickle cell anemia (SCA) is one of the most common inherited monogenic diseases in the world, affecting more than 300,000 newborns annually [1]. It is estimated that 50–90% of SCA infants born in Sub-Saharan Africa, where the disease is most prevalent, die before the age of 5 years [2]. Most of these SCA children in low-income countries suffer from acute infections responsible for worsening anemia, such as malaria and invasive pneumococcal disease, considered leading causes of death in this group [2,3]. Due to the high morbidity and mortality rates, SCA is considered a global public health concern [4].

The carriers of the sickle cell trait (HbAS) are often asymptomatic, whereas homozygotes (HbSS) suffer from SCA, which is the most severe form of sickle cell disease (SCD) [5,6]. These patients have a lifelong disorder mainly characterized by chronic hemolytic anemia, vaso-occlusive crisis (VOC), strokes, pulmonary hypertension, and bacterial susceptibility that can lead to septicemia [1,2,4]. However, infants typically only start to experience SCA symptoms after 6 months of age, when HbF levels begin to decrease [7]. Given the global population growth, which is faster in tropical and subtropical regions, demographic projections suggest that the annual number of births affected by SCA will increase by 33% until 2050 [5]. Although the burden of this disorder is increasing, there are only a few therapeutic strategies available.

Until very recently, hydroxyurea (HU) was the only FDA- and EMA-approved drug for the treatment of patients with SCA [1]. It is considered a ribonucleotide reductase inhibitor that, when administered once daily at the correct dose (normally 15–30 mg/kg), increases fetal hemoglobin levels (HbF); however, its magnitude of response is very variable [8,9].

The Pediatric Hydroxyurea Phase 3 Clinical Trial (BABY HUG), which was a placebo-controlled clinical trial performed in SCA infants, revealed that HU administration was associated with significantly lower rates of initial and recurrent episodes of pain, dactylitis, acute chest syndrome, and hospitalizations [10]. The results obtained demonstrate the safety of use in patients 9 months of age and older. However, the authors of this clinical trial did not expect gastroenteritis to be also significantly less frequent in SCA children taking HU since it has not previously been reported in other studies [10]. Additionally, in the same study, all the infections caused by *Streptococcus pneumoniae* occurred in the placebo group, suggesting that hydroxyurea may have a protective role against pneumococcal bacteremia/sepsis [10]. This same hypothesis was already proven in another study with a sickle cell mouse model of pneumococcal pneumonia and sepsis treated with HU [11]. These authors were able to demonstrate that hydroxyurea decreased circulating leukocytosis, reduced leukocyte recruitment to the infected lung, reduced invasion of bacteria into the bloodstream, decreased sE-selectin levels in serum, and significantly improved the survival from pneumococcal infection [11].

Considering all these studies, it is unquestionable that HU has a protective effect, not only by improving several hematological parameters but also by lowering the risk of some bacterial infections. However, the direct impact of this type of treatment on the human microbiome has not yet been investigated.

The microbiome is involved in many vital bodily functions from helping host digestion and vitamin synthesis to supporting the immune system development and preventing infections [12]. When the balanced interaction between the gastrointestinal tract and resident microbiota is disrupted (dysbiosis), some diseases may develop or aggravate, such as inflammatory bowel disease, chronic kidney disease, atherosclerosis, cardiovascular disease, obesity, allergies, asthma, hypertension, and coronary artery disease [13]. Furthermore, it has been demonstrated that the host’s genotype and immune system significantly impact the development of gut microbiota [13].

In fact, recent studies have reported pathophysiologic and microbial changes in the gut of SCD patients, including enterocyte injury, altered microbial composition, increased permeability, and bacterial overgrowth [14]. One evidence that supports the microbiome involvement in SCD, comes from the observation that these patients have considerably higher numbers of circulating aged neutrophils compared with healthy controls, and when receiving antibiotics or rifaximin, these numbers decrease, associated with a reduction of VOC episodes [15,16]. Since intestinal microbes are the main regulators of aged neutrophils, it is hypothesized that SCD patients have a chronic disequilibrium of the intestinal microbiome or abnormalities in the integrity of the intestinal barrier, which can result in recurrent bacterial translocation and dysbiosis, contributing to the higher levels of circulating aged neutrophils observed [15,17].

Moreover, it is believed that microbial modulation by dietary intervention or direct supplementation (with specific nutrients or probiotics) may be an effective strategy for preventing cardiovascular diseases, either alone or in conjunction with existing therapies [18]. However, before fully relying on the approaches targeting gut modulation, the interactions between gut microbial communities, HU effect, neutrophils, and VOC frequency, which remain an important unanswered question influencing SCA, should be explored in detail.

The aim of this work was to assess the effect of HU administration on the gut microbiome composition of an Angolan pediatric population with SCA and compare it with the clinical, hematological, and biochemical parameters.

## 2. Results

### 2.1. Overview of Clinical Characteristics

This longitudinal study included 33 SCA Angolan children eligible for hydroxyurea treatment, aged between 4–12 years old (mean 8.15 ± 2.11). This group of patients comprised both genders (63.6% females).

The clinical laboratory characteristics of the SCA children before and after 6 months of HU treatment are shown in Table 1. As expected in hydroxyurea treatments, several hematological and biochemical parameters changed, with significant increases registered in total hemoglobin, fetal hemoglobin, MCV, and MCH, as well as reductions in WBC, neutrophils, reticulocytes, and total bilirubin, demonstrating compliance to the treatment. Moreover, hydroxyurea treatment reduced the rates of painful events and the number of hospitalizations and transfusions per year.

### 2.2. Metagenomic Data

Stool samples from 33 individuals before and 6 months after initiation of HU treatment were collected, giving 66 samples sequenced for the V3-V4 regions of bacterial 16S rRNA. After quality trimming and chimera checking, a total of 3,671,944 high-quality processed sequences were obtained, with an average of 50,301 valid reads per sample and a mean length of 242 bp. High coverage values (average = 99.5%) were obtained for sequences in all samples, indicating a good sequencing depth. In total, 4109 operational taxonomic units (OTUs) were identified, with an average of 1644 OTUs per sample. If we subdivide the population into two groups, before and after HU, the OTUs average is 2095 and 1192, respectively. Sequences were then classified at each phylogenetic level from phylum to species.

### 2.3. Hydroxyurea Impact on Alpha and Beta Diversities

**Figure 1:**
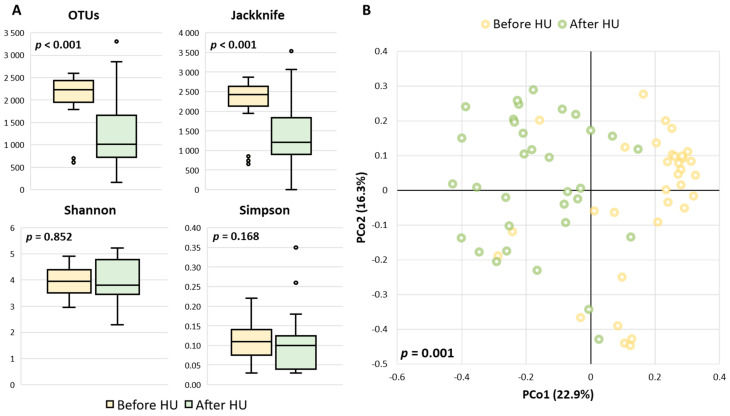
(**A**) Boxplots of operational taxonomic units (OTUs) observed and species richness (Jackknife index) and diversity performed with Shannon and Simpson indexes. Alpha-diversity analysis was conducted using the Wilcoxon test; (**B**) Principal-coordinate analysis (PCoA) based on Bray–Curtis distances at genus level showing a clustering pattern between the two groups. Beta-significance was calculated by the PERMANOVA test, and the percentage of variation explained by the principal coordinates (PCo1 and PCo2) is indicated on the axes.

The alpha and beta diversity profiles before and after HU treatment were quite distinct. The number of observed OTUs and indexes for species richness (Ace, Chao1, Jackknife) indicated statistical differences with the Wilcoxon test (*p* < 0.001), suggesting a higher diversity before the initiation of hydroxyurea (Figure 1A). As for the estimates of species evenness (Shannon and Simpson indexes), no significant differences were observed between the two groups.

For beta-diversity analysis, the microbial community structure was visualized using Principal Coordinates Analysis (PCoA) with Bray-Curtis’s index (Figure 1B). The graph revealed a sharp segregation along PCo1, which represents 22.9% of the inter-sample variance, indicating a strong core division between the communities before and after HU treatment. This difference in bacterial genera communities was statistically significant, as determined with the permutational multivariate analysis of variance of distance matrices (PERMANOVA, *p* = 0.001).

### 2.4. Hydroxyurea-Induced Differences in Gut Microbiome Composition

**Figure 2:**
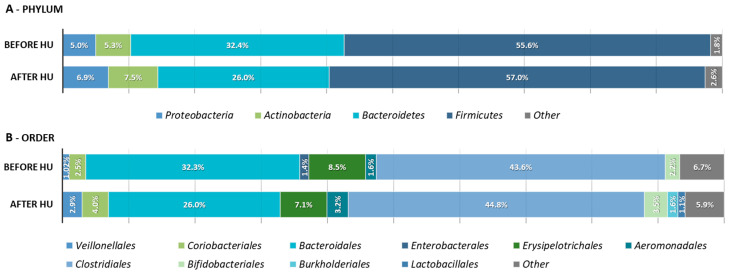
(**A**) Phylum-level and (**B**) Order-level composition of the microbial community after sequencing. Bacteria with a relative abundance of less than 1% were grouped as “Other”.

**Figure 3:**
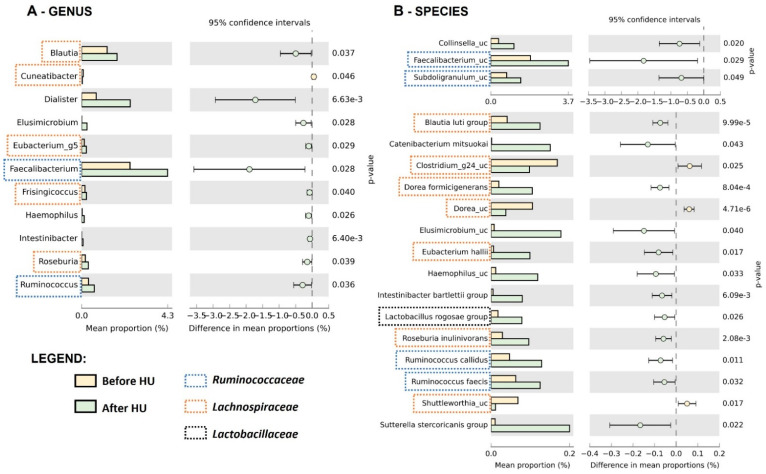
Distribution of the mean proportions of bacterial abundances between SCA children before (*n* = 33) and after (*n* = 33) HU treatment, regarding the taxonomic rank of (**A**) genus and (**B**) species. Besides the patient’s group, the legend indicates three relevant taxonomic families: *Ruminococcaceae, Lachnospiraceae*, and *Lactobacillaceae*. The results were filtered using a *p*-value lower than 0.05 with Welch’s *t*-test, the effect size of 0.05 threshold, and retaining unclassified reads in STAMP software.

Next, we evaluated specific microbial features that could be associated with HU treatment. A comparison of relative abundances in children before and after hydroxyurea revealed multiple differences at different taxonomic levels. The most abundant phyla were Firmicutes, followed by Bacteroidetes, Actinobacteria, and Proteobacteria (Figure 2A). Bacteroidetes were more prevalent before HU (*p* = 0.019) at the phylum level, which led to a significant difference in the F/B ratio (*p* = 0.033). As for the genus level (Figure 3A), only *Cuneatibacter* was more prevalent before HU (*p* = 0.046), whereas individuals after treatment had higher numbers of *Blautia*, *Dialister*, *Elusimicrobium*, *Eubacterium_g5*, *Faecalibacterium*, *Frisingicoccus*, *Haemophilus*, *Intestinibacter*, *Roseburia*, and *Ruminococcus*. In Figure 3B, it is possible to observe that, after HU, several species were more predominant: *Blautia luti*, *Catenibacterium mitsuokai*, *Collinsella_uc*, *Dorea formicigenerans*, *Elusimicrobium_uc*, *Eubacterium halli*, *Faecalibacterium_uc*, *Haemophilus_uc*, *Intestinibacter bartlettii*, *Lactobacillus rogosae*, *Roseburia inulinivorans*, *Ruminococcus callidus*, *Ruminococcus faecis*, *Subdoligranulum_uc*, and *Sutterella stercoricanis*. Moreover, only three species had a higher abundance before HU: *Clostridium_g24_uc*, *Dorea_uc*, and *Shuttleworthia_uc*.

## 3. Discussion

Given that drugs are among the strongest factors influencing the gut microbial community, by changing the intestinal microenvironment or affecting microbial metabolism [19], it was expectable that after the intake of hydroxyurea this population would exhibit some microbiota differences. Our first findings supported this claim with statistical differences observed in species richness (Ace, Chao1, Jackknife) and beta-diversity indexes among the two groups. However, no significant results were found in the indexes for species evenness (Shannon and Simpson). This is probably because the richness indexes from alpha-diversity take more into account the OTUs, which were already statistically significant in our sample. In contrast, Shannon and Simpson are considered estimators of taxa diversity, combining both taxa richness and evenness within organisms, measuring the distribution of abundances instead of only measuring the number of taxonomic groups [20].

Hydroxyurea is considered an antineoplastic antimetabolite drug and was initially used as a chemotherapeutic agent until its benefits for SCA treatment were recognized more than 30 years ago [9]. It is reported that most of the antineoplastic antimetabolites decrease the alpha diversity of gut microbiota [21], which was consistent with our results. However, the same study also reported that most of these drugs can also induce gut microbiota dysbiosis, particularly the imbalance of the F/B ratio, and reduce the SCFA-producing gut species, probably due to the toxicity induced [21]. This was not in agreement with our results, but it is important to take into consideration that this population suffers from a chronic disease and the clinical benefits brought by hydroxyurea administration could surpass the disadvantages/adverse effects in the majority of cases. In fact, several hematologic and biochemical parameters improved after HU, demonstrating not only compliance to the treatment but also drug efficacy for this SCA population.

Commensal intestinal bacteria can play a key role in alleviating inflammation and decreasing the risk of bacteremia [22]. The children after hydroxyurea had higher numbers of several beneficial bacteria such as *Blautia* spp., *Blautia luti*, *Ruminococcus* spp., *Ruminococcus callidus*, *Ruminococcus faecis*, *Eubacterium_g5*, *Eubacterium halli*, *Faecalibacterium* spp., *Roseburia* spp., *Roseburia inulinivorans*, and *Subdoligranulum* spp. In the literature, *Ruminococcus callidus*, *Ruminococcus albus*, *Blautia obeum*, and *Prevotella* spp. are considered fiber-degrading bacteria with the ability to produce solubilized oligosaccharides and polysaccharides that act as substrates for butyrate-producing species such as *Faecalibacterium prausnitzii, Eubacterium rectale, Roseburia* spp., *Subdoligranulum* spp., *Eubacterium hallii*, and *Anaerostipes* spp. [18,22,23]. This is especially important because microbes that ferment essential short-chain fatty acids (SCFAs), such as butyrate, are usually responsible for maintaining microbial homeostasis by stimulating the production of mucin, antimicrobial peptides, tight-junction proteins and reducing colonic oxidative stress [22,24]. The lack of butyrate can silence important metabolic signaling in the gut, resulting in a transfer of oxygen which allows for pathogenic facultative anaerobes, such as *E. coli*, to outcompete the benign microbes [24].

Besides these beneficial bacteria, *Lactobacillus rogosae* and *Elusimicrobium* were also more prevalent after hydroxyurea. The *Lactobacillus* bacteria are essential for the maintenance of intestinal barrier function, providing an inhibitory environment to the growth of many pathogenic bacteria with the production of lactic acid and also exerting anti-inflammatory effects by the secretion of lactocepin [25,26]. *Elusimicrobium* is described as a potential beneficial bacteria thought to be involved in inflammatory cytokine regulation, being negatively correlated with IL-6 and IL-17 [27].

In the Firmicutes phylum, the OTUs from the *Ruminococcaceae*, *Lactobacillaceae*, *Lachnospiraceae* family were frequently more abundant after HU. In particular, several members within *Lachnospiraceae* family (*Blautia, Dorea, Roseburia, Eubacterium, Cuneatibacter, Frisingicoccus,* and *Shuttleworthia*), most of them commonly present in the human intestine, showed significant differences in our population. Jenq et al. [28] demonstrated that *Lachnospiraceae* family was associated with decreased lethality from graft-versus-host disease after allogenic blood/marrow transplantation and higher amounts of *Blautia* improved the overall survival in the same study.

In a previous paper from our group, we reported that *Blautia* was less prevalent in SCD children than in the control group [29]. Interestingly, in this study, both *Blautia* spp. and *Blautia luti* increased in the same SCA population after 6 months of hydroxyurea. Not only *Blautia*, but also *Roseburia* and *Eubacterium hallii* are often associated with a healthy state. A detailed review [30] mentioned that these same bacteria are main SCFAs producers involved in the butyril-CoA:acetate CoA trasferase pathway, with a number of studies evidencing their beneficial effects, such as strengthening of the intestinal barrier, inflammation modulation, reinforced insulin sensitivity and glucose metabolism, decreased circulating lipid plasma levels and maturation of the immune system. Moreover, *R. inulinivorans*, *B. obeum*, and *E. hallii* also regulate the propanediol pathway and *D. formicigenerans* the mucin degradation, both pathways have propionate as an end product of bacterial metabolism, which is another SCFA [30]. A recent study demonstrated that propionate had beneficial anti-inflammatory properties limiting cardiovascular disease development since it was able to attenuate cardiac hypertrophy, fibrosis, vascular dysfunction, and hypertension in two different mouse models [31].

Although the children after HU had a higher frequency in several beneficial bacteria, there were some exceptions, namely with *Intestinibacter bartlettii*, *Dialister* spp., and *Haemophilus* spp. There are only a few studies that address the effects of *Intestinibacter bartlettii*, formerly called *Clostridium bartlettii*. One of the studies discovered a positive correlation between anorexia and fatigue in recovered healthcare workers with COVID-19 [32], and another study found that these bacteria were more frequently present in patients with neurodevelopmental disorders [33]. As for *Dialister* species, some of them (*D. pneumosintes* and *D. invisus*) have been identified as pathogens, mainly associated with oral infections, such as periodontitis and acute necrotizing ulcerative gingivitis, but their relative significance in human clinical samples remains unknown [34].

It is well known that SCD children are normally at increased risk for bloodstream infections due to impaired or absent splenic function, immune defects, and anatomic predisposition as occurs in osteomyelitis [35]. A retrospective cohort study of SCD children performed several blood cultures (19,902 in total) from 2010 to 2019 and discovered that the most common pathogens in this population were *Streptococcus pneumoniae*, *Streptococcus viridansgroup*, *Escherichia coli*, *Staphylococcus aureus*, *Bordetella holmesii*, *Haemophilus influenzae*, and *Salmonella species* [35]. In this study, no statistical differences in *Staphylococcus, Salmonella*, and *Streptococcus* genera were observed. As for the *Haemophilus*, we noticed that only three children after HU had a proportion greater than 0.5% for this genus, being possible outliers for this comparison.

One of the few bacteria more prevalent in the group before HU was an unclassified *Clostridium_g24*, a group that includes *C. aldenense, C. asparagiforme, C. lavalense, C. clostridioforme, C. citroniae*, and *C. bolteae.* The last one, *C. bolteae*, is commonly present in stools of children, but its overabundance can be responsible for chronic diarrheal episodes [36]. Moreover, both *C. bolteae* and *C. clostridioforme* are considered opportunistic pathogens, which have been isolated from intra-abdominal infections, bacteremia, and even abscesses [36].

The composition and metabolic ability of the gut microbiome have a major role in pharmacokinetics and could determine drug toxicity and clinical efficacy through its impact on chemical transformation [19,21,37]. Conversely, drugs can also change the microbial community composition and function by altering the intestinal microenvironment, microbial metabolism, or bacterial growth [37]. Since the microbiome is highly individualized, different patients may have distinct responses to the absorption, distribution, and metabolism of certain drugs [19]. On the other hand, this also provides a significant potential for biomarkers to predict drug responses, and correct dosage and could optimize a certain therapy, promoting a more personalized approach [21].

Our study had some limitations, as we were only able to analyze 33 Angolan children before and after treatment. Future studies with another geographic population or with a different age range could obtain different results given the high impact of environment, nutrition, and age on the gut microbiome. The factors which could regulate or influence the gut microbiota baseline should be included in a follow-up research analysis. These comparisons are undoubtedly more indicated for longitudinal studies that track microbiome changes over time in the same population. Although, a major problem with these types of studies is the loss of subjects, which happened in this project since we started with 40 individuals.

Overall, our results suggest that hydroxyurea can influence the diversity and shape of the gut microbiome, implying that interactions between SCA phenotype, HU, and intestinal bacteria are important. However, it is not yet clear if the higher abundance of beneficial bacteria is a direct or indirect effect of HU treatment, being a consequence rather than a cause. It is possible that the overall improvement of the inflammatory state in SCA caused by HU, in terms of reduction of neutrophils, VOC crises, or higher HbF levels, could mitigate the intestinal dysbiosis frequently experienced by these patients.

**Figure 4:**
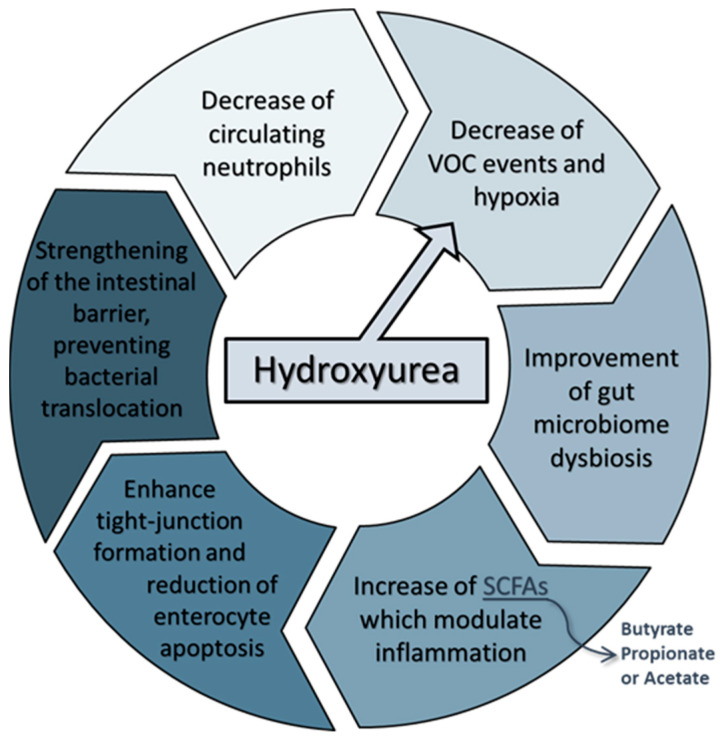
Illustration of the hydroxyurea impact on the gut microbiome of SCA patients.

Dutta et al. [38] described how SCD patients display an altered intestinal microbiome due to the intermittent hypoxia and hypoxemia induced by microvascular obstruction from the recurrent VOC crises. These changes in the gut microbiome composition can decrease the production of SCFAs and disrupt tight-junction formation and enterocytes apoptosis, leading to bacterial translocation across the intestinal barrier into the systemic circulation to stimulate neutrophils [15,38]. Given this, we speculate that the higher abundance of SCFAs producing species observed after HU intake could be enhancing tight-junctions formation and restoring the intestinal barrier of these patients, which may be decreasing the translocation of intraluminal bacteria and bacterial products that stimulate inflammation. The significant decrease of neutrophils in this population group also supports this hypothesis (Figure 4). Taken all together, intestinal dysbiosis and bacterial translocation are possibly contributing to the higher levels of circulating neutrophils, and the reduction of VOC crises due to HU intake is positively altering the gut microbiome, leading to the higher production of SCFAs and the reduction of neutrophils.

## 4. Materials and Methods

### 4.1. Study Design and Ethical Considerations

We conducted a longitudinal study between October 2020 and October 2021, recruiting a total of 33 patients previously diagnosed with SCA from 2 different hospitals in Angola, located in the province of Bengo at “Hospital Geral do Bengo” and in the city of Luanda at “Hospital Pediátrico David Bernardino”. The only inclusion criteria were no recent antibiotic exposure prior to sample collection and an acceptance of HU treatment. All patients were examined by a pediatrician in regular consultations who obtained the clinical details.

Since all participants were minors, their legal guardians provided written informed consent for study participation. The participants’ guardians were informed of the study’s objectives as well as the option to withdraw at any time without further obligation. The study was reviewed and approved by the Ministry of Health of Angola and the Ethical Committee of ESTeSL, in compliance with the guidelines of the Helsinki Declaration.

### 4.2. Sample Collection and Clinical Analyses

Sample collection was performed prior to, and 6 months after, hydroxyurea treatment, and included a whole blood sample for hematological and biochemical analysis, and stool samples for bacterial microbiome analysis. All the children were naïve for HU. To ensure sample stability during transportation and storage, the fecal samples were collected using the DNA/RNA Shield Fecal Collection tubes (Zymo Research, Irvine, CA, USA).

The XT-2000i Hematology Analyzer (Sysmex Corporation, Kobe, Japan) was used for hematological determinations that comprised complete blood count (erythrocytes, reticulocytes, white blood cells, and platelets), hemoglobin, mean corpuscular volume (MCV), and mean corpuscular hemoglobin (MCH). The fetal hemoglobin fraction was quantified by HPLC (Biorad Variant II, Hercules, CA, USA). The biochemical analysis included Lactate dehydrogenase (LDH), creatinine, urea, total, and direct bilirubin, Aspartate Aminotransferase (AST) and Alanine Aminotransferase (ALT) determinations, Cobas C111 (Roche Diagnostics, Basel, Switzerland), and Mindray BA-88A (Mindray, Shenzhen, China).

### 4.3. DNA Extraction and 16S rRNA Sequencing

Microbial DNA was extracted using ZymoBIOMICS™ DNA Miniprep Kit (Zymo Research, Irvine, CA, USA) and FastPrep-24™ homogenizer (MP Biomedicals, Strasbourg, France) according to the manufacturer’s instructions. The NanoDrop One spectrophotometer (Thermo Fisher Scientific, Waltham, MA, USA) was used to quantify and evaluate the purity of the DNA samples, which were then stored at −20 °C until further processing.

Preparation of libraries for sequencing was done following the 16S Metagenomic Sequencing Library Preparation Illumina document, in which we used the KAPA HiFi HotStart Ready Mix (Roche) and primers to amplify V3-V4 hypervariable regions of the bacterial 16S rRNA gene [39]. Following PCR amplification, the dsDNA HS assay kit for the Qubit 3.0 fluorometer (Thermo Fisher Scientific, Waltham, MA, USA) and the High Sensitivity D1000 ScreenTape and Reagents for TapeStation 4200 (Agilent Technologies, Santa Clara, CA, USA) were used to determine DNA concentration and amplicon lengths, respectively. Illumina index adapters were added for each sample and all purification steps were carried out using AMPure XP magnetic beads (Beckman Coulter, Brea, CA, USA). The resulting indexed libraries were once again checked on TapeStation and quantified on Qubit. Then, an equimolar pool of 4 nM was prepared for further denaturation, dilution to 2 pM, and sequencing on the NextSeq550 instrument (Illumina, San Diego, CA, USA) with 2 × 151 bp paired-end reads. After this procedure, the software generated analysis output into FASTQ files format.

### 4.4. Quality-Control and Statistical Analysis

Microbiome taxonomic profiles were generated using the EzBioCloud MTP pipeline and EzBioCloud 16S database PKSSU4.0 [40]. The single-end reads were uploaded in this software for merging paired-end reads, trimming primers, data quality checking, and filtering out sequences of low quality regarding read length (<100 bp or >2000 bp) and averaged Q values less than 25. This pipeline also used the UCHIME algorithm to check and remove chimeras. All sequences that did not match any reference with at least a 97% similarity cutoff were clustered using the UCLUST method with a 97% cutoff.

Statistical analysis for clinical data and alpha-diversity indexes were conducted using the non-parametric Wilcoxon test with IBM SPSS Statistics version 27.0 (IBM). These include species richness estimators (such as ACE, CHAO, and Jackknife) and diversity indices (Shannon and Simpson). For beta-diversity, a Principal Coordinate Analysis (PCoA) was performed using the Bray-Curtis dissimilarity index to evaluate the microbiota structure of the different population groups. Differences between the microbiota composition at various taxonomic levels were assessed through Welch’s *t*-test using the Statistical Analysis of Metagenomic Profiles (STAMP) software package v2.1.3 [41]. *p*-values < 0.05 were considered statistically significant.

## 5. Conclusions

This study provides the first evidence of the HU effect on the gut microbiome and unravels several OTUs, mainly SCFAs producing species, that could be considered candidate biomarkers for this disease and HU efficacy or even potential probiotics targets. In this context, more studies aiming to understand the underlying mechanism of how hydroxyurea and SCD change the microbiome, and which preexisting bacteria could lead to better treatment response, are unquestionably needed and should help to clarify some of the outstanding questions. Further studies aiming to evaluate the impact of other SCD drugs and resulting metabolites should also be considered. This will not only support the reproducibility of our findings but will also contribute to characterizing the SCD microbiome and optimize treatment efficacy in the near future.

## Figures and Tables

**Table 1:** Hematologic and biochemical characteristics of the SCA children before and after the hydroxyurea treatment (*n* = 33).

Variables	Before HU		After HU		*p*-Value *
	Mean	±SD	Mean	±SD	
Hemoglobin (g/dL)	7.36	±0.89	7.68	±0.93	**0.031**
Fetal Hemoglobin (%)	4.67	±3.85	7.58	±4.79	**<0.001**
Erythrocytes (10^12^ L)	2.93	±0.57	2.71	±0.47	**0.001**
MCV (fL)	77.33	±6.86	84.61	±8.72	**<0.001**
MCH (pg)	25.53	±2.53	28.93	±3.63	**<0.001**
WBC (10^9^ L)	12.92	±3.73	9.23	±3.11	**<0.001**
Neutrophils (10^9^ L)	5.46	±1.65	3.64	±1.96	**<0.001**
Platelets (10^9^ L)	432.61	±169.08	394.79	±164.77	0.249
Reticulocytes (%)	10.26	±4.13	7.58	±3.62	**<0.001**
LDH (U/L)	523.84	±151.11	691.22	±215.07	**0.001**
Creatinine (mg/dL)	0.65	±0.75	0.53	±0.30	0.970
Total Bilirubin (mg/dL)	1.83	±1.93	1.09	±0.85	0.096
Direct Bilirubin (mg/dL)	0.93	±1.38	0.61	±0.56	0.544
Urea (mg/dL)	32.88	±15.95	29.65	±15.90	0.357
AST (U/L)	33.07	±17.33	47.94	±15.71	**0.001**
ALT (U/L)	11.78	±10.47	19.70	±10.75	**0.001**

* Wilcoxon test. MCV: mean corpuscular volume, MCH: mean corpuscular hemoglobin, WBC: white blood cells, LDH: lactate dehydrogenase, AST: aspartate aminotransferase, ALT: alanine aminotransferase, SD: standard deviation.

## Data Availability

The datasets generated during and/or analyzed during the current study are available from the corresponding author on reasonable request.
